# Structural and Binding Properties of Two Paralogous Fatty Acid Binding Proteins of *Taenia solium* Metacestode

**DOI:** 10.1371/journal.pntd.0001868

**Published:** 2012-10-25

**Authors:** Seon-Hee Kim, Young-An Bae, Hyun-Jong Yang, Joo-Ho Shin, Sylvia Paz Diaz-Camacho, Yukifumi Nawa, Insug Kang, Yoon Kong

**Affiliations:** 1 Department of Molecular Parasitology, Sungkyunkwan University School of Medicine and Center for Molecular Medicine, Samsung Biomedical Research Center, Suwon, Korea; 2 Department of Microbiology, Graduate School of Medicine, Gachon University, Inchon, Korea; 3 Department of Parasitology, Ewha Womans University, School of Medicine, Seoul, Korea; 4 Division of Pharmacology, Department of Molecular Cell Biology, Sungkyunkwan University School of Medicine and Center for Molecular Medicine, Samsung Biomedical Research Center, Suwon, Korea; 5 Facultad de Ciencias Químico Biológicas, Universidad Autónoma de Sinaloa, Culiacán, Sinaloa, México; 6 Faculty of Medicine, Khon Kaen University, Khon Kaen, Thailand; 7 Department of Molecular Biology and Biochemistry, School of Medicine, Kyung Hee University, Seoul, Korea; McGill University, Canada

## Abstract

**Background:**

Fatty acid (FA) binding proteins (FABPs) of helminths are implicated in acquisition and utilization of host-derived hydrophobic substances, as well as in signaling and cellular interactions. We previously demonstrated that secretory hydrophobic ligand binding proteins (HLBPs) of *Taenia solium* metacestode (TsM), a causative agent of neurocysticercosis (NC), shuttle FAs in the surrounding host tissues and inwardly transport the FAs across the parasite syncytial membrane. However, the protein molecules responsible for the intracellular trafficking and assimilation of FAs have remained elusive.

**Methodology/Principal Findings:**

We isolated two novel *TsMFABP* genes (*TsMFABP1* and *TsMFABP2*), which encoded 133- and 136-amino acid polypeptides with predicted molecular masses of 14.3 and 14.8 kDa, respectively. They shared 45% sequence identity with each other and 15–95% with other related-members. Homology modeling demonstrated a characteristic β-barrel composed of 10 anti-parallel β-strands and two α-helices. TsMFABP2 harbored two additional loops between β-strands two and three, and β-strands six and seven, respectively. TsMFABP1 was secreted into cyst fluid and surrounding environments, whereas TsMFABP2 was intracellularly confined. Partially purified native proteins migrated to 15 kDa with different isoelectric points of 9.2 (TsMFABP1) and 8.4 (TsMFABP2). Both native and recombinant proteins bound to 11-([5-dimethylaminonaphthalene-1-sulfonyl]amino)undecannoic acid, dansyl-DL-α-amino-caprylic acid, *cis*-parinaric acid and retinol, which were competitively inhibited by oleic acid. TsMFABP1 exhibited high affinity toward FA analogs. TsMFABPs showed weak binding activity to retinol, but TsMFABP2 showed relatively high affinity. Isolation of two distinct genes from an individual genome strongly suggested their paralogous nature. Abundant expression of TsMFABP1 and TsMFABP2 in the canal region of worm matched well with the histological distributions of lipids and retinol.

**Conclusions/Significance:**

The divergent biochemical properties, physiological roles and cellular distributions of the TsMFABPs might be one of the critical mechanisms compensating for inadequate *de novo* FA synthesis. These proteins might exert harmonized or independent roles on lipid assimilation and intracellular signaling. The specialized distribution of retinol in the canal region further implies that cells in this region might differentiate into diverse cell types during metamorphosis into an adult worm. Identification of bioactive systems pertinent to parasitic homeostasis may provide a valuable target for function-related drug design.

## Author Summary

Neurocysticercosis (NC), an infection of the central nervous system with *Taenia solium* metacestode (TsM), constitutes a leading cause of adult-onset seizures in endemic areas. Like other helminths, TsM is incapable of synthesizing lipid molecules. It should be equipped with a specialized system for lipid transportation from the host to ensure its long-survival. Such a transport system may be a target for function-associated drug design. We characterized two novel fatty-acid (FA)-binding TsM proteins (TsMFABP1 and TsMFABP2). Native and recombinant proteins bound to several FA analogs and retinol at micromolar and millimolar concentrations. Their binding was specifically inhibited by oleic acid. TsMFABP1exhibited high affinity toward FA analogs, while TsMFABP2 showed preferential affinity to retinol. Both TsMFABPs were predominantly expressed in the canal region of the worm, where lipids and retinol were abundantly distributed. The two paralogous TsMFABPs have undergone (or are still undergoing) structural diversification and following functional divergence to act as FABP or retinol binding protein, similar to the intracellular lipid binding proteins of deuterostomian animals. The canal region specific distribution of lipids, retinol and FABPs further suggested that cells in this area might differentiate into diverse cells to compose huge numbers of the proglottids, thereby playing vital roles in the parasite growth and development.

## Introduction

Neurocysticercosis (NC), caused by infection of the central nervous system (CNS) with *Taenia solium* metacestode (TsM), represents one of the most common CNS helminthic diseases and invokes formidable public health problems. NC is associated with several neurological manifestations including seizure, headache and focal neurologic deficits, which may vary according to the location, number and viability of the parasites within the brain [1]. NC is endemic worldwide, but is more prevalent in Latin America, the Indian subcontinent, Sub-Saharan regions and Southeast Asian countries, where approximately 50 million people are at risk of infection. NC has been increasingly detected in developed countries due mainly to immigrants from endemic areas [2], [3]. The clinical aspects, neuroimaging and serodiagnosis of NC have been relatively well characterized [4 and references therein]. However, the functional aspects of the pathogen including cellular biochemical and molecular mechanisms inherent to the maintenance of cellular homeostasis have largely remained elusive.

Parasitic helminths exploit limited lipid metabolism due to low levels or an absence of enzymes involved in the oxygen-dependent pathway. They depend mostly on essential lipids imported from their host and have evolved special hydrophobic ligand binding systems to ensure their long-survival in the harsh, low-oxygen tension host environments [5]. A series of lipid binding proteins have been characterized from the platyhelminths. The hydrophobic ligand binding proteins (HLBPs) are small α-helix rich 7–10 kDa molecules with extremely hydrophobic binding site(s). Their functions included uptake and storage of the hydrophobic molecules, and cellular protection by lowering free fatty acid (FA) concentrations below toxic levels [6], [7]. Some of these molecules, especially those of TsM, are reliable serodiagnostic biomarkers for NC [4], [8], [9]. No orthologous protein has been identified in other organisms. The molecules form a novel cestode-specific HLBP family [10] showing unique properties including oligomer/multimer formation in normal physiological conditions [11].

FA binding proteins (FABPs) are cytosolic proteins of approximately 15 kDa. They have been implicated in intracellular uptake, transport and storage of hydrophobic ligands, regulation of lipid metabolism and sequestration of excess toxic FAs [12], as well as in signaling and regulation of gene expression [13], [14]. The proteins bind non-covalently to hydrophobic ligands, especially to FAs and retinol. They belong to the intracellular lipid binding protein (iLBP), which comprises the calycin superfamily, together with the avidin and lipocalin families. The family members show varying degrees of sequence identity among the members (approximately 15–70%), but conserve a characteristic β-barrel structure, which consists of 10 anti-parallel β-strands and two α-helices [15]. During the course of chordate evolution, an ancestral iLBP gene has diverged into three subfamilies of FABP, cellular retinoic acid binding protein (CRABP) and cellular retinol binding protein (CRBP), after gene duplication [16]. Each of the protein subfamilies is subdivided into several isoforms with tissue-specific distribution and function in vertebrates [17].

Several FABPs from platyhelminths including *Echinococcus granulosus*, *Mesocestoides vogae* (syn. *M. corti*), *Schistosoma* spp. and *Fasciola* spp. have been characterized [18]–[25]. These proteins display structural and biochemical properties similar to vertebrate orthologs, especially the human heart-type FABP [26]. They are not only involved in trafficking, storage/utilization of intracellular FA and protection of several intracellular enzymes from the detergent effects of FAs, but also in inducing antibody responses and protective immunity in the hosts [25], [26]. Although the crystal structure of *E. granulosus* FABP1 has been elucidated [27], highly limited information is available regarding structural/functional diversification and tissue specificity of FABPs in platyhelminths.

Our previous *ex vivo* experiments with viable TsMs demonstrated that secretory TsM HLBP shuttles FAs in the surrounding host tissues and conveys them into the parasite across the biological barrier [11], while downstream molecules responsible for the intracellular trafficking and assimilation of the transported FAs have not been elucidated. In this study, we isolated two novel genes encoding FABPs and investigated their biochemical and functional properties, which might act as the intracellular counterparts of the HLBP by mediating intracellular transportation of hydrophobic molecules.

## Materials and Methods

### Ethics Statement

All animals used in this study were housed in accordance with guidelines from the Association for the Assessment and Accreditation of Laboratory Animal Care (AAALAC). All protocols were approved by the Institutional Review Board and conducted in the Laboratory Animal Research Center of Sungkyunkwan University (protocol 2006-02-048) and Universidad Autónoma de Sinaloa, Mexico (2008).

### Parasite Samples

TsMs were collected from naturally infected pig in Sinaloa state, Mexico. Intact worms were individually collected and washed with physiological saline >10 times. Cyst fluid (CF) was collected as previously described [11]. The whole worm, scolex and neck, and bladder wall were separately homogenized with a Teflon-pestle homogenizer in phosphate buffered saline (PBS; 100 mM, pH 7.2) supplemented with protease inhibitor cocktail (1 tablet/25 ml; Complete; Roche). The CF and homogenates were centrifuged for 1 h at 20000 *g*. The supernatants were employed as crude CF and the respective extracts. Thirty fresh worms were incubated in 25 ml RPMI 1640 (Gibco) supplemented with the protease inhibitor cocktail for 1 h at 37°C. Addition of protease inhibitor cocktail into culture medium did not induce harmful effects on the excretory-secretory products (ESP) [28]. The incubation medium was harvested and centrifuged at 500 *g* for 10 min followed by 20000 *g* for 1 h. The supernatants were used as ESP. All procedures were done at 4°C unless otherwise specified. Samples were stored at −80°C until use.

### Isolation of TsM Genes Encoding FABPs

We previously constructed a TsM cDNA library using the lambda Uni-ZAP system and determined the nucleotide sequences of the randomly picked clones from 5′-regions with the universal T3 promoter primer [29]. We selected two clones, designated *TsMFABP1* and *2*, which showed significant degrees of sequence identity with numerous FABPs during BLAST analysis of the GenBank databases at the NCBI (http://www.ncbi.nlm.nih.gov/). The TsM cDNA library was screened by polymerase chain reaction (PCR) using vector (T3 and T7 promoter primers) and *TsMFABP*-specific primers (sense, 5′-GGCACGAGGATCAGATCGGGTGGTC-3′ and antisense, 5′-AGAGGGCGCTTTTGTATTTCACGTC-3′ for *TsMFABP1*; and sense, 5′-TAATTAACCCTCACTAAAGGGAAC-3′ and antisense, 5′-AAAAGGTGTCAAAGTGGGCTTGTTG-3′ for *TsMFABP2*). T3 promoter primer and the antisense primers were used to amplify the 5′-regions of the respective genes. The sense primer and T7 promoter primer were employed to amplify their 3′-regions. The thermal cycler profile included preheating at 94°C (2 min), 35 cycles at 94°C (40 sec), 60°C (30 sec) and 72°C (1 min) with a final extension at 72°C (10 min). Amplicons were ligated into the pGEM-T Easy vector (Promega) and sequenced using the ABI Prism Dye Terminator Cycle Sequencing Core Kit (Perkin Elmer) and a Bioapply 3730 XL automated DNA sequencer (Perkin Elmer). In order to increase the accuracy of nucleotide sequences, we used the high fidelity *Pfu* DNA polymerase (Clontech) during the PCR amplification and determined them from both strands of five clones. Contig cDNAs were obtained by overlapping the 5′- and 3′-region sequences. Their integrity was further confirmed by PCR using primers matched to each terminus of the contig sequences. The genomic structures were determined by amplifying each of the homologous DNAs from genomic DNAs extracted from a single worm. The genomic sequences were aligned with their corresponding mRNA sequences by considering the exon-intron boundary sequences, after which their chromosomal structures were determined.

### Structural Prediction of TsMFABPs

The coding profiles and homology patterns were analyzed with the ORF Finder and BLAST programs (NCBI). A search for the functionally and structurally conserved protein domains was conducted using ProfileScan (http://myhits.isb-sib.ch/cgi-in/motif_scan). The secondary structures were predicted by PDH software. The tertiary structures were predicted by comparative modeling method by ESyPred3D (http://www.fundp.ac.be/sciences/biologie/urbm/bioinfo/esypred/) using the *E. granulosus* FABP1 (Protein Data Bank id. 1O8V; 95% identity) as a template and visualized with PyMol [30] as a template. The quality of predicted tertiary models was further evaluated by calculating template modeling score (TM-score) and root mean square deviation (RMSD) between TsMFABPs and other related proteins with the I-TASSER program (http://zhanglab.ccmb.med.umich.edu/I-TASSER/), which combined the methods of threading, *ab initio* modeling and structural refinement [31].

### Phylogenetic Analysis

In order to retrieve the closely matched sequences from a variety of GenBank genomic databases, the deduced amino acid (aa) sequences of *TsMFABPs* were used as queries in the BLAST searches. A total of 168 sequences were selected by considering both the homology values and taxonomical distributions. Human proteins representing distinct subfamilies of iLBP were additionally retrieved from the databases. The aa sequences of two data sets were separately aligned with ClustalX and optimized using GeneDoc. The alignments were used as inputs to analyze the phylogenetic relationships among the members with MEGA program (ver4.0). The sequence divergences were calculated with the Jones-Taylor-Thornton (JTT) substitution model and indels between pairs of sequences were regarded as missing data. The phylogenetic trees were constructed by the neighbor-joining algorithm. The statistical significance of each branching node was evaluated employing 1000 random samplings of the input alignments by the SEQBOOT program.

### Expression and Purification of Recombinant TsMFABPs (rTsMFABPs)

The cDNAs corresponding to the predicted ORF region of TsMFABPs were PCR-amplified with specific primers containing cleavage sites for restriction enzymes (underlined) of *Bam*HI and *Xho*I (*TsMFABP1*, 5′-CGCGGATCCATGGAGCCATTCATCG-3′ and 5′-TGACTCGAGTTACGCTGCCTTAAC-3′; *TsMFABP2*, 5′-GCGGATCCATGACCTCAAGTGAG-3′ and 5′-CACTCGAGTCAGCTCTTCTGCCG-3′) and directionally cloned into the pET-28a expression vector (Novagen) following enzyme digestion. The plasmids were transformed into *Escherichia coli* BL21 (DE3) (Novagen). Each of single colonies containing the insert was used to initiate a liquid culture and expression of recombinant protein was induced by 0.5 mM isopropyl β-D-1-thiogalactopyranoside (IPTG). The recombinant proteins were purified from bacterial lysates by Ni-NTA affinity chromatography using a HiTrap chelating Sepharose column (Amersham Biosciences). The recombinant proteins were monitored by 15% reducing SDS-PAGE with Coomassie Brilliant Blue (CBB) G-250 staining.

### Generation of Mouse Antibodies against Recombinant Proteins (anti-rTsMFABPs)

Polyclonal antisera against each recombinant protein were raised in specific pathogen-free, 6-week-old female BALB/*c* mice by consecutive subcutaneous inoculation of the respective proteins (30 µg) in Freund's adjuvant at 2-week intervals. A final booster was done by intravenous injection of 10 µg/100 µl PBS without adjuvant through tail vein. One week later, blood was collected by cardiac puncture. The immune sera were obtained by centrifugation at 3000 *g* for 10 min. IgG fractions were isolated using a Protein G affinity chromatography column (Amersham Biosciences) and stored at −80°C until required.

### Purification of Native TsMFABPs

TsM whole worm extracts were fractionated by a Superdex 75 prep grade (HiLoad, 16×60 cm-long) molecular sieve fast protein liquid chromatography (FPLC) system (AKTA; Amersham Biosciences), which was equilibrated with Tris-HCl (20 mM, pH 8.0) containing 150 mM NaCl. The extracts (10 mg proteins/3 ml) were applied to the column (flow rate; 0.5 ml/min) and 85 fractions (each 1.5 ml aliquot) were allocated according to their absorbance at 280 nm monitored by UNICORN (ver3.0). Fractions showing high lipid-binding activity with concomitant positive reactions against anti-rTsMFABPs were pooled, dialyzed against Tris-HCl (20 mM, pH 9.0) and concentrated. Ion-exchange chromatography was further conducted on a 2×10 cm-long DEAE-Sepharose column (Amersham Biosciences) equilibrated with Tris-HCl (20 mM, pH 9.0). Elution was done with a step-wise gradient NaCl concentrations (0, 20, 40, 60, 80 and 100 mM) with the same buffer. Active fractions identified as above were dialyzed against 20 mM Tris-HCl (pH 8.0) containing 150 mM NaCl, concentrated and stored at −80°C until use.

### SDS-PAGE, Two-Dimensional Electrophoresis (2-DE) and Immunoblotting

Respective TsM extracts (10 µg) and rTsMFABPs (100 ng) were resolved by 15% SDS-PAGE under reducing conditions. For 2-DE, partially purified TsMFABPs (10 µg) were mixed with rehydration buffer (6 M urea, 2 M thiourea, 2% CHAPS, 0.4% dithiothreitol [DTT], 0.5% IPG buffer and 0.002% bromophenol blue [BPB]), loaded on IPG strips (pH 6–11) with a cup-loading instrument (IPGphor; Amersham Biosciences) and focused for a total of 35 kVh. Second-dimension SDS-PAGE was done by 15% gels (160×160×1 mm). The separated proteins were visualized with CBB G-250 or transferred to nitrocellulose (NC) membranes (Schleicher & Schuell). The membranes were blocked for 1 h with Tris buffered saline (100 mM, pH 8.0) containing 0.05% Tween 20 and 5% skim milk (blocking buffer), after which they were incubated overnight with specific mouse antibodies (1∶2000 dilutions) in blocking buffer. The membranes were incubated with a 1∶4000 dilution of horseradish peroxidase (HRP)-conjugated goat anti-mouse IgG (Cappel) for an additional 1 h. The reactions were developed with an enhanced chemiluminescence (ECL) kit (Pierce). For quantitative analysis, all the immunoblot images were developed after 1 min exposure.

### Fluorometric Ligand Binding Assay

All the proteins were delipidated for 2 h using Sephadex-LH (Sigma-Aldrich) prior to assay. The ligand binding profile of the native and rTsMFABPs were detected spectrofluorometrically using fluorescent FA analogs, including 11-([5-dimethylaminonaphthalene-1-sulfonyl]amino)undecannoic acid (DAUDA), dansyl-DL-α-aminocaprylic acid (DACA) (Molecular Probes), retinol (Sigma-Aldrich) and naturally fluorescent *cis*-parinaric acid (cPnA; Molecular Probes). Fluorescence emission spectra were recorded at 25°C with a total volume of 200 µl per well using black 96-well Microfluor 1 plates and an Infinite M-200 automated multi-detector (Tecan). The emission and excitation wavelengths for DAUDA, DACA, retinol and cPnA were 519, 519, 325 and 420 nm, and 345, 350, 350 and 315 nm, respectively. We included the TsM 120-kDa protein (10 µM) and recombinant 18 kDa (5 µM; a subunit of the TsM 120-kDa protein), which were proven not to have FA-binding activity [32], as negative controls during the measurements. All fluorescent stock compounds (10 mM dissolved in ethanol) were stored at −20°C in a dark room and were freshly diluted in ethanol prior to use. The equilibrium dissociation constants (*K*_d_) of the proteins bound to DAUDA, retinol and cPnA were estimated by adding increasing concentrations of respective ligands (0.1–10 µM for FA analogs and 0.1–10 mM for retinol) in a micro-quartz plate. Fluorescence intensities were normalized to the peak fluorescence intensity and corrected for background fluorescence of the ligand alone at each concentration. Corrected data were analyzed using the one-site saturation model and best fit algorithm contained in SigmaPlot9 software (*y* = *V*_max_ *X*/*K*_m_+*X*, where *y* is relative fluorescence and *X* is concentrations of lipid ligand. *V*_max_ can be substituted as *F_max_* [maximum fluorescence]). Competition assays were carried out by monitoring the change in fluorescence intensity at the peak transmission wavelength measured for either rTsMFABPs:DAUDA, rTsMFABPs:retinol or rTsMFABPs:cPnA complex in the presence of 10-fold excess oleic acid.

### Nile Red Staining and Retinol Autofluorescence

Fresh TsMs were evaginated in the presence of 1% bile salts (Sigma-Aldrich) in RPMI 1640 (pH 7.2) at 37°C overnight. The worms were fixed in 4% paraformaldehyde in PBS (50 mM, pH 7.4) at 4°C, dehydrated with a graded alcohol and embedded in paraffin. Sections 4 µm in thickness were cut, deparaffinized and rehydrated. A stock solution was prepared by dissolving Nile red (9-diethylamino-5H-benzo[α]phenoxazine-5-one, 100 µg/ml; Sigma-Aldrich) in acetone and stored at −20°C in the dark until use. The stock solution (10 µl) was diluted in 70% glycerol (10 ml) just prior to use. A drop of diluted Nile red solution was placed on the fresh TsM sections for 1 h at 4°C. The slides were mounted on Paramount Aqueous mounting medium (DAKOCytomation) and observed using a LSM510 Meta DuoScan confocal microscope (Carl Zeiss). The locality of retinol (vitamin A) was observed on the 10 µm-thick cryosectioned TsM sections under an Axioplot light/fluorescent microscope (excitation filter BP365/12, barrier filter BP495/40; Carl Zeiss) [33]. Since treatment of worm sections with organic solvent removed retinol and the biochemical was quickly oxidized when exposed to the air, unfixed and unstained frozen sections were observed immediately after mounting.

### Immunohistochemical Staining and Fluorescence *in situ* Hybridization (FISH)

The tissue distribution of TsMFABPs was determined on evaginated worm sections using the respective antibodies. Worm sections (4 µm-thick) were treated with 3% hydrogen peroxide for 5 min and blocked with PBS supplemented with 3% bovine serum albumin (BSA) and 0.05% Tween 20 (PBS/T-BSA) for 1 h. The sections were incubated with the respective antibodies (1∶200 dilutions in PBS/T-BSA) overnight at 4°C. For fluorescent staining, rhodamine-conjugated goat anti-mouse IgG antibody (Jackson) was incubated for 1 h at 4°C. The slides were counterstained with 4′,6-diamidino-2-phenolindole (DAPI, 10 µg/ml; Invitrogen) for 5 min at 4°C in dark and observed under an Axioplot light/fluorescent microscope (Carl Zeiss). Pre-immune mouse serum diluted to the same ratio was employed as a control.

*In situ* hybridization was conducted using fluorescent Cy5-labeled probes (TsMFABP1 anti-sense, 5′-CGCTGCCTTAACGTAGGTTCGCACGC-3′ and sense, 5′-GCGTGCGAACCTACGTTAAGGCAGCG-3′; TsMFABP2 antisense 5′-GCTCTTCTGCCGACGGTACATGTGCAC-3′and sense 5′-GTGCACATGTACCGTCGGCAGAAGAGC-3′). The worm cryosections mounted on superfrost PLUS slides (Sigma-Aldrich) were rehydrated in 10% formamide and 2× SSC for 5 min, followed by treatment with proteinase K. The hybridization reactions were performed in hybridization solution (100 µl) for 16 h at 55°C. The slides were then washed with washing buffer (20% formamide in 2× SSC) 2 times for 30 min at 30°C. Nuclear staining was done by adding DAPI (Invitrogen) to the wash solution during the second wash. The slides were mounted with freshly prepared oxygen depleted mounting media. The signals were observed using a LSM510 Meta DuoScan confocal fluorescence microscope (Carl Zeiss).

## Results

### Molecular Characteristics of Two Novel TsMFABP Genes

Similarity analyses of TsM expressed sequence tag clones against the GenBank database and following cDNA library screening led to the identification of two full-length cDNAs, which displayed high structural similarity with the other known FABPs. The TsM genes, designated *TsMFABP1* and *TsMFABP2*, encoded an ORF for 133- and 136-aa polypeptide with predicted molecular masses of 14.3 and 14.8 kDa and isoelectric point (pI) values of 8.6 and 8.4, respectively. The coding regions shared 48% and 45% identity with each other at the nucleotide and aa levels, respectively. The initial BLASTX searches with the *TsMFABP* sequences at the NCBI retrieved several hundred FABPs isolated from diverse organisms. They showed the highest matches to those of cestode parasites including *E. granulosus* and *M. vogae* (identity >53% and E-value <3e-28 for *TsMFABP1*, identity >42% and E-value <9e-18 for *TsMFABP2*). Homology searches by the Hidden Markov models revealed the results similar to those with BLAST algorithms (data not shown).

**Figure 1:**
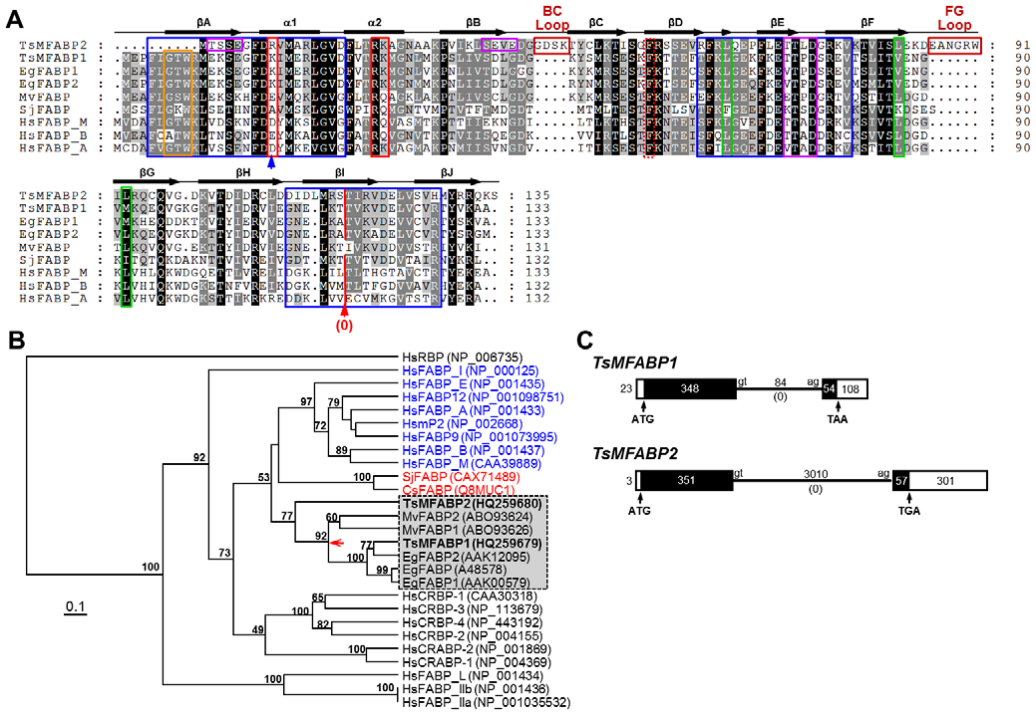
Molecular properties of TsMFABPs. (**A**) The primary structures of TsMFABP1 and TsMFABP2 s were compared with those of related members. Dots represent gaps introduced into the sequences to increase similarity values. The secondary structure predicted with the TsMFABP1 sequence is shown at the top of the alignment. Brown boxes denoted as BC and FG loops indicate amino acid (aa) extensions found uniquely in TsMFABP2. Motifs 1, 2 and 3 are marked by blue boxes. The GXW triplet domain in motif 1 is shown by orange box. The aa residues involved in nuclear localization signal and its regulation site are shown by red and dotted red boxes, respectively. The hormone-sensitive lipase binding site is indicated by a blue arrow. Residues comprising the nuclear export signal are denoted by green boxes. Purple boxes indicate the putative signal sequences targeted for the phosphorylation. Red vertical line marked with a red arrow represents the position of orthologous introns conserved among the related proteins. TsMFABP1, HQ259679; TsMFABP2, HQ259680; *E. granulosus* FABP1 (EgFABP1), AAK00579; EgFABP2, AAK12095; *M. vogae* FABP (MvFABP), ABO93626; *Schistosoma japonicum* FABP (SjFABP), CAX71489; human muscle FABP (HsFABP_M), CAA39889; human brain FABP (HsFABP_B), NP_001437; human adipocyte FABP (HsFABP_A), NP_001433. (**B**) Phylogenetic positions of TsMFABPs and their platyhelminth orthologs were predicted among the human intracellular lipid binding family members such as FABP (_E, epidermis; _H, heart; _I, intestine; _L, liver; _Ila/b, ileal isoform a and b; mP2, myelin P2) and cellular retinol-/retinoic acid-binding protein (CRBP/CRABP). The tree was constructed using the neighbor-joining algorithm of MEGA and rooted with human plasma retinol binding protein (HsRBP). The statistical significance of the branching nodes (numerical in each of the nodes) was estimated by a bootstrapping analysis of 1000 replicates of initial input alignment. An internal node connecting major cestode proteins is indicated by a red arrow. CsFABP, *Clonorchis sinensis* FABP. (**C**) The genomic structures of *TsMFABP1* and *TsMFABP2* were determined from chromosomal segments homologous to each of the respective cDNAs. The lengths of predicted exons (rectangles) and introns (solid lines) are shown in base pairs. The open rectangles indicated the 5′- and 3′-untranslated regions. The initiation (ATG) and stop (TAA/TGA) codons in the open reading frame, as well as the nucleotides found in the exon-intron boundary (gt-ag), are marked in the corresponding positions. The intron phases are shown in parentheses.

The primary structures of TsMFABPs were compared with those of some cestode and human orthologs. As shown in Figure 1, these molecules revealed variable degrees of sequence identity from 44%–95%, but tightly conserved several signatures and motifs representative of the FABP family. Motifs 1, 2, and 3, spanned the βA-α1 (23 aa), βE (17 aa) and βI–βJ (22 aa) domains (blue boxes). Nuclear localization signal with three basic aa residues was positioned at K18/R9, R30/21 and K31/22, and its regulation site was found at F58/62, respectively (red and dotted red boxes). Nuclear export signal was observed at L60/62, V82/L82 and M92/L92 (green boxes). Hormone-sensitive lipase binding site was recognized at K18/R9 (blue arrow) (positions of respective aa residue denote each for TsMFABP1 and 2). The GXW triplet, which is shared by the members of calycin superfamily, was found in the motif 1 (orange box), but the TDY triplet found in the lipocalin family was not detected in the motif 2 of TsMFABPs and related proteins. Interestingly, TsMFABP2 contained two aa insertions between βB and βC (4 aa, BC loop), and between βF and βG (6 aa, FG loop). In addition, TsMFABP1 conserved a single site for protein kinase C and casein kinase II phosphorylation, while TsMFABP2 harbored three sites targeted for the casein kinase II phosphoylation (purple boxes).

The tertiary structures of TsMFABPs were readily simulated using the *E. granulosus* FABP1 (Protein Data Bank id. 1o8vA) as a template during homology-based modeling. The models were highlighted by the basic β-barrel composed of 10 anti-parallel β-strands (βA–βJ) and N-terminal helix-turn-helix motif (α1 and α2) (Figure S1). The extra loops detected in TsMFABP2 were placed near the bottom of the barrel (pinkish boxes). A similar structure for TsMFABP1 protein was predicted by different threading templates such as 1o8vA, 3rswA and 1hmsA by I-TASSER program (confidence score 1.43, TM-score 0.91±0.06, RMSD 1.8±1.5 Å). The I-TASSER result with TsMFABP2 sequence was similar to that of TsMFABP1, while the quality of predicted model seemed to be less significant, due probably to the extra BC and FG loops (confidence score 0.50, TM-score 0.78±0.10, RMSD 3.6±2.5 Å). We deposited nucleotide sequence data under the accession numbers HQ259679 (*TsMFABP1*) and HQ259680 (*TsMFABP2*) in the GenBank database.

### Phylogenetic Analysis

A phylogenetic tree constructed with the aa sequences of 168 TsMFABP-related proteins demonstrated different clustering patterns between the protostomian and deuterostomian FABPs (Figure S2). The proteins isolated from the invertebrates were closely allocated to one another according to the taxonomical positions of their donor organisms, whereas those from higher animals appeared to be split into several monophyletic sub-clades containing each of the iLBP families, regardless of their donor sources. The relative phylogenetic positions of TsMFABPs were further examined against diverse human iLBP members (Figure 1B). A neighbor-joining tree placed these platyhelminth proteins between the human myelin-adipocyte-heart FABP and the CRBP/CRABP subfamilies, suggesting that the platyhelminth proteins have not yet been differentiated into each of the subfamily lineages. The TsMFABP1 was interconnected to other cestode proteins by an internal node (red arrow in Figure 1B), while TsMFABP2 comprised a single external node. The trematode proteins formed a clade separated from that of cestode homologs. The trees constructed using the maximum-likelihood (TREE_PUZZLE) and maximum-parsimony (PHYLIP) algorithms also showed a tree topology similar to that of neighbor-joining method (data not shown).

The genomic structure of *TsMFABP* genes was determined employing the genomic DNA extracted from a single worm. The genomic sequences of *TsMFABPs* contained a single intron of 84-bp (*TsMFABP1*) or 3010-bp (*TsMFABP2*) near the 3′-end of the respective ORFs. The intron was located prior to the first nucleotide of a codon (phase 0) within both TsM genes (Figure 1C). The intron appeared to be orthologous among the related genes used in the phylogenetic analysis, except for the *M. vogae* (*MvFABP*s) and out-group gene (*HsRBP*), despite the great length polymorphism (red vertical line with a red arrow, Figure 1A). This result suggested that the paralogous *TsMFABP1* and *2* genes have arisen by duplication of an ancestral gene at least before divergence of cestode species.

### Purification and Ligand Binding Specificities of TsMFABPs

The bacterially expressed recombinant proteins were purified by Ni-NTA affinity chromatography. The rTsMFABPs migrated to approximately 18 kDa, which were slightly larger (3 kDa) than that predicted by the aa sequences, due to the additional N-terminal tag (Figure S3A). We also partially purified the native TsMFABPs through gel filtration followed by DEAE anion-exchange chromatography. TsMFABP1 and 2 were eluted at flow-through and 20 mM fractions, respectively. When these proteins were analyzed by 2-DE and subsequent immunoblotting probed with each of the specific antibodies, a single immunoreactive signal at 15 kDa and a pI value of ca. 9.2 (TsMFABP1) or 8.4 (TsMFABP2) was detected (Figure S3B).

**Figure 2:**
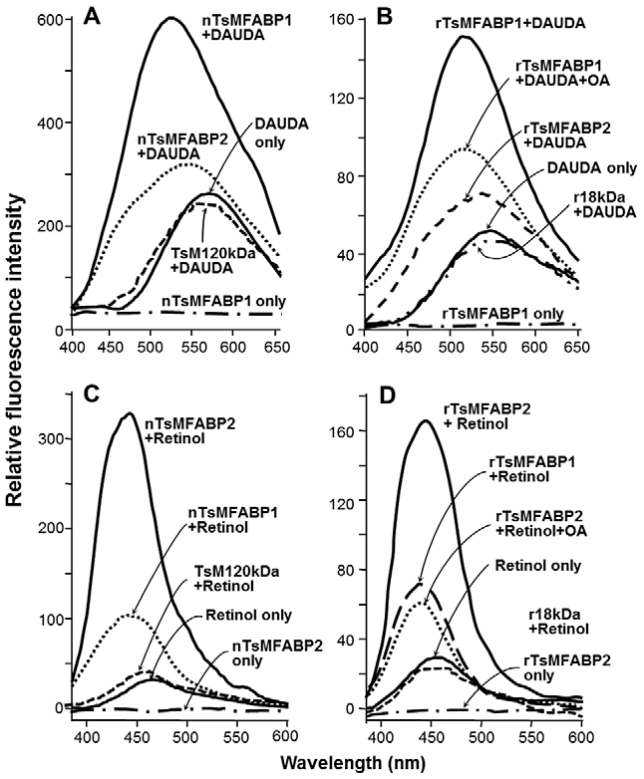
*In vitro* binding activity of native and recombinant TsMFABPs against DAUDA and retinol. Fluorescence emission spectra of DAUDA (0.1 µM, Ex_max_ = 345 nm) (**A** and **B**) and retinol (5 mM, Ex_max_ = 350 nm) (**C** and **D**) bound to the purified native (nTsMFABPs) (**A** and **C**) and recombinant (rTsMFABPs) (**B** and **D**) proteins (each 1 µM) were recorded at 25°C (200 µl/well) using a black 96-well micro-Fluor plate. DAUDA (10 mM stock dissolved in ethanol) was stored −20°C in darkness and were freshly diluted in ethanol just before use. The competitive binding of oleic acid (OA, 5 µM) to the performed rTsMFABP1:DAUDA or rTsMFABP2:retinol complex is also shown (**B** and **D**). TsM 120-kDa (TsM120 kDa, 10 µM) and recombinant 18-kDa (r18 kDa, 5 µM) proteins were included in the assay as negative controls.

The partially purified native and recombinant proteins were subjected to delipidation. Each of the proteins (1 µM) was used in a hydrophobic ligand binding assay against the polarity-sensitive fluorophore-tagged FA analogs (0.1 µM) and retinol (5 mM). The fluorescence emission of DAUDA was significantly increased with a blueshift from 550 nm to 500 nm, when mixed with the native or rTsMFABP1 (Figures 2A and 2B) indicating the engagement of fluorophore into a highly non-polar DAUDA binding site. The interactive binding was competitively inhibited by oleic acid in a dose-dependent manner (Figure 2B, part of data not shown). The TsMFABP2 also bound to DAUDA, although its specific activity was lower than that of the TsMFABP1. Both of the TsM proteins exhibited binding affinity toward retinol. Interestingly, the relative activities were reversed when retinol was provided as the hydrophobic ligand (Figures 2C and 2D). The retinol-binding activity of rTsMFABP2 appeared to be higher than that of rTsMFABP1. Other fluorescent FA analogs such as DACA and cPnA showed interaction modes comparable to those with DAUDA against the rTsMFABPs (data not shown). No binding activity was detected in the reactions with the TsM 120-kDa and recombinant 18-kDa proteins, which were used as negative controls.

The steady-state kinetics of binding reactions assayed using rTsMFABPs demonstrated saturation behavior in accordance with the increasing concentrations of DAUDA and cPnA (0.1–10 µM), and retinol (0.1–10 mM). The dissociation constants (*K*_d_) of rTsMFABP1 were calculated to be 2.15 µM, 0.28 µM and 1.78 mM for DAUDA, cPnA and retinol, respectively, whereas the equivalent values for rTsMFABP2 were determined to be 9.40 µM, 0.64 µM and 0.98 mM, respectively. The binding rate constants (*V_max_*/*K*_d_) against each of the hydrophobic ligands were also highly distinguishable between the rTsMFABP1 and rTsMFABP2: 139.3 *versus* 38.7 for DAUDA, 2172.9 *versus* 483.0 for cPnA and 172.1 *versus* 211.3 for retinol (Table 1).

**Table 1:** Comparison of kinetic parameters for the binding reaction of the rTsMFARs against DAUDA, retinol and *cis*-parinaric acid (cPnA).

Ligand	Parameter	recTsMFABP1	recTsMFABP2
DAUDA	*V*max^a^	299.5±3.0	364.2±3.5
	*K*_d_ (µM)	2.15±0.14	9.40±0.89
	*V*max/*K*_d_	139.3±12.7	38.7±3.94
cPnA	*V*max	608.4±57.8	309.1±28.4
	*K*_d_ (µM)	0.28±0.02	0.64±0.04
	*V*max/*K*_d_	2172.9±214.6	483.0±47.1
Retinol	*V*max	306.3±31.4	207.1±19.6
	*K*_d_ (mM)	1.78±0.15	0.98±0.08
	*V*max/*K*_d_	172.1±15.5	211.3±20.3

a The rate was calculated by measuring relative fluorescence of each reaction.

### Histological Distribution of Native TsMFABPs

**Figure 3:**
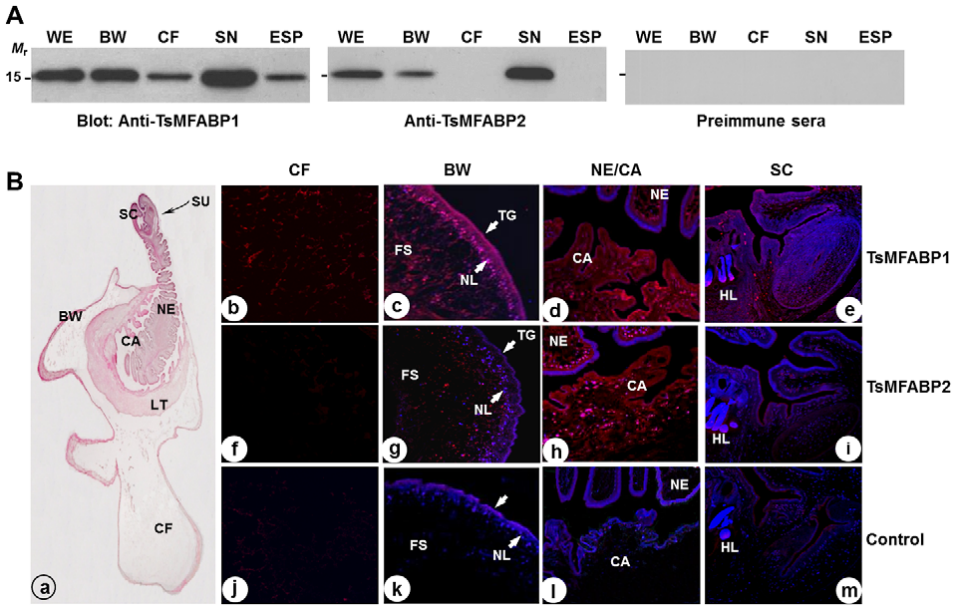
Expression patterns of TsMFABPs. (**A**) Proteins (each of 5 µg) extracted from the whole TsM (WE), bladder wall (BW), scolex and neck (SN), and cyst fluid (CF) were separated by 15% SDS-PAGE under reducing conditions, after which processed with immunoblotting probed with the respective antibodies or a preimmune mouse serum (1∶2000 dilutions). Proteins contained in the excretory-secretory products (ESP) were also included. The blots were developed with an ECL system. *M*_r_, molecular mass in kDa. (**B**) Tissue locality of TsMFABPs was examined in the TsM sections by immunohistochemical staining. The whole image of evaginated worm stained with hematoxylin-eosin is shown (a). Worm sections were incubated with mouse antibodies specific to rTsMFABPs or a preimmune serum (1∶200 dilutions) and then, with rhodamine-conjugated anti-mouse IgG antibody. The slides were counterstained with DAPI (10 µg/ml). CA, spiral canal; FS, fibrillar stroma of BW; HT, hooklet; LT, loose tissue; NE, neck; NL, nuclear layer of BW; SC, scolex; SU, sucker.

We examined tissue expression pattern of TsMFABPs employing soluble TsM proteins extracted from different anatomical compartments. TsMFABPs were expressed in the TsM parenchyme including scolex and neck, and bladder wall, although that of TsMFABP2 appeared relatively low in the bladder wall. Interestingly, the CF and ESP proteins, where the excretory-secretory proteins accumulate, reacted with the antiserum specific to rTsMFABP1, but not with that against rTsMFABP2. The same blot probed with preimmune mouse serum did not exhibit any response (Figure 3A).

**Figure 4:**
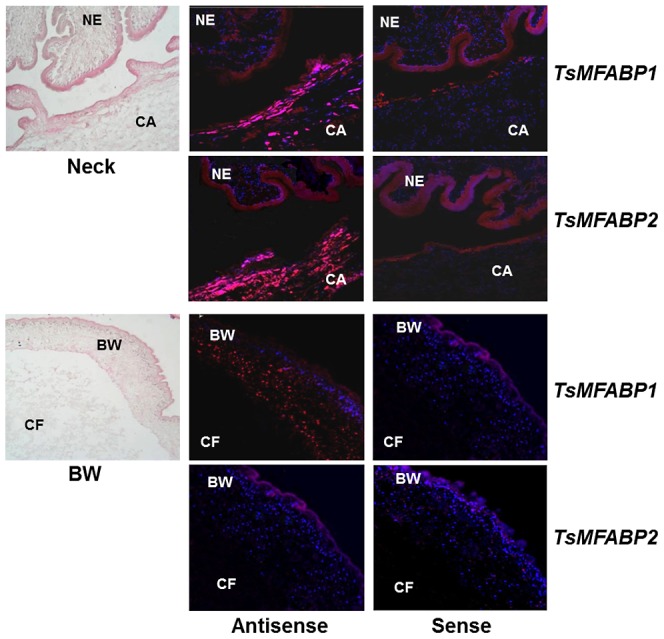
Expression of mRNA transcripts of *TsMFABP* genes. Fresh TsM sections were probed with Cy5-labeled, *TsMFABP*-specific antisense or sense probes, after which counterstained with DAPI (10 µg/ml). Positive signals for *TsMFABP* expressions are shown in bright-pinkish color. BW, bladder wall; CA, spiral canal; CF, cyst fluid; NE, neck.

The histological distribution of TsMFABPs was further examined on the TsM sections by immunohistochemical staining. Figure 3B (panel a) presents an evaginated worm section stained with hematoxylin-eosin, in which characteristic tissues/organs of TsM including scolex, neck, spiral canal, loose tissue and bladder wall were observed. These two proteins exhibited principally similar anatomical distribution in the worm section, but some variable pattern was also recognized. Anti-rTsMFABP1 antibody mainly reacted with protein(s) scattered in the bladder wall and spiral canal. The signal appeared to be prominent in the subtegumental nuclear layer and the spherical cell body-like compartments scattered through the fibrillar stroma of the bladder wall (panel c). The antibody revealed a similar reaction pattern in the spiral canal. In the neck, the reaction intensity was relatively weak and was largely restricted in the nuclear layer zone (panel d). In contrast, protein(s) in CF showed fairly weak positive reactions, which suggested that small amount of TsMFABP1 are secreted into surrounding environments. The scolex did not exhibit any detectable reaction (panel e). The TsMFABP2 was intensely localized in the subtegumental regions and relatively less in the stroma beneath the subtegumental nuclear layer of the neck and spiral canal (panel h). The bladder wall revealed weak positive reactions (panel g), while CF and scolex did not show any detectable signal (panels f and i). The expression patterns observed at the protein levels matched well with the results obtained by *in situ* hybridization, in which each transcript was stained with Cy5-labeled, gene-specific antisense probes on the TsM cryosections (Figure 4). Both transcripts created high signals at spiral canal region.

### Distribution Patterns of Lipids and Retinol in the TsM Sections

**Figure 5:**
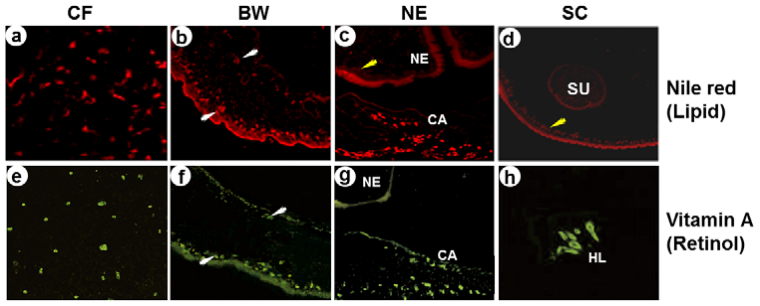
Tissue distribution of lipids and retinol in the TsM. The evaginated worm sections were stained with Nile red for visualization of hydrophobic lipids and observed under fluorescent microscope (**a–d**). The sections were observed under fluorescence microscope to detect the green autofluorescent characteristics of retinol (**e–h**). Yellow arrows indicate subtegumental regions of neck and sclolex. White arrows indicate the inner and outer surfaces of bladder wall. CF, cyst fluid; BW, bladder wall; CA, spiral canal; HL, hooklet; NE, neck; SC, scolex; SU, sucker.

Lipid molecules are shuttled by HLBPs/FABPs during the intracellular trafficking. We analyzed the tissue distribution of lipid droplets and retinol in the worm sections. As shown in Figure 5 (panels a–d), the lipid droplets stained with Nile red were primarily distributed within the bladder wall and spiral canal in a scattered fashion, and less in the subtegumental regions of the neck and scolex (yellow arrows). The distribution density of the droplets was found to be irregular across the bladder wall. Strong signals were detected in the outer surface regions of the bladder wall membrane (white arrows), whereas the inner regions were faintly stained with the hydrophobic dye. Nile red was also stained with some hydrophobic droplets/molecules in the region filled with CF, which has been suggested to act as a reservoir for lipid molecules taken from host environments [11]. Retinol, when exposed and excited with ultraviolet light, emits a natural, green fluorescence and faded away within 20 sec [34]. Retinol was largely restricted in the outer membranous region of the spiral canal and bladder wall compared to those of Nile red-positive molecules (Figure 5, panels f and g). Retinol was also detected in CF (panel e), although no significant signal could be observed by the anti-rTsMFABP2 antibody (Figure 5, panel f). Hooklets showed non-specific epifluorescence (panel h)

## Discussion

FAs are highly versatile and heterogeneous compounds, which play essential roles in the construction of cell membrane, energy metabolism, glycoprotein synthesis and signaling pathway associated with cellular interactions and proliferation [35]. Retinoids share a number of characteristics with FAs including biochemical formula during storage, circulation and function, as well as physicochemical properties. The hydrophobic FAs and retinoids bind to iLBPs such as FABP and CRBP/CRABP, which have diverged from a common ancestor to gain characteristic aa residues and/or structural motifs that bestow highly specialized functions in vertebrates [36]. Except for few cases, invertebrate iLBPs display significant similarities with the FABP group [13], [26], while an understanding of physiological functions and evolutionary episode of iLBP family largely remains elusive in the lower animal taxa including cestode parasites [24 and references therein].

In this study, we identified two novel paralogous TsM proteins that share domain organization, motifs and functional aa residues characteristic to the intracellular FABPs of metazoan animals [37]. When we assessed the ligand binding activity of native and recombinant TsMFABPs, their binding affinity toward FA analogs was much greater than that against retinol (Table 1). TsMFABP1 was broadly localized in the fibrillar stromal region of the bladder wall in addition to the spiral canal zone, but TsMFABP2 showed more restricted distribution pattern in the canal region surrounding the neck. These results suggest that TsMFABP1 might be a counterpart of the TsM 150-kDa HLBP to relay the trafficking of FAs in the intracellular phase [11]. The presence of TsMFABP1 in intracellular and extracellular compartments further supports the notion that the protein might be involved not only in the uptake of host FAs from the surrounding environments, to cooperate with or to compensate to the 150-kDa protein function, but also in the storage of exogenous hydrophobic molecules, thus acting as a genuine intracellular FABP (Figure 3). As we could not detect classical signal peptidase recognition site by the PSORT (http://psort.nibb.ac.jp) and SignalP (http://www.cbs.dtu.dk/services/SignalP) programs, the protein might be secreted through a signal peptide-independent mechanism [38]. On the other hand, TsMFABP2 might have evolved, or is still evolving, to acquire a novel property to operate as a retinol transporter, if not all, in certain circumstances, such as high retinol concentrations.

TsMFABP1 and 2 showed typical hydrophobic ligand binding activity with the sizeable dissociation constants in 10^−6^ M range, like the FABP members characterized in other organisms including cestode parasites [13], [14], [24], [27], while these proteins revealed relatively weak binding activity toward retinol (*K*_d_ values within 10^−3^ M). Since other cestode FABPs/HLBPs currently characterized had no retinol binding activity, retinol binding protein(s) and biological roles of retinol in cestode physiology largely to be determined. However, tissue distribution of TsMFABP2 in the subtegumental regions of the spiral canal surrounding the neck (Figure 5) correlated well with that of retinol. The competitive binding of oleic acid with TsMFABP2 resulted in significant replacement of retinol from the binding site (Figure 2). Homology modeling of TsMFABPs revealed tertiary structures similar to those of the other FABP members, except for two extra loops found in TsMFABP2. These collective data suggest that the interactive binding affinity between TsMFABPs and retinol, especially that of TsMFABP2, is specific, although the binding affinity is notably low compared to other mammalian CRBPs. During diversification, TsMFABPs might gain additional ligand binding activity toward retinol.

It is generally accepted that when TsMs are ingested by humans, the neck and surrounding tissues constitute an active growing portion for the metamorphosis. The posterior portion of the neck is a starting point for development of long and numerous segments of the adult worm. Therefore, provision of huge amount of various bioactive molecules including FA and retinol in this area might be crucial for the continuous generation of rapidly maturing numerous proglottids, which contain several essential resources for growth and development of the reproductive systems. It is highly adventurous that during the development of the vertebrates, retinol is abundantly and specifically distributed in the posterior position, which is a niche for cellular differentiation through the transcriptional regulation of several specific genes [39] and functions as a signaling molecule [40]. Similar histological distribution of TsMFABP2 and retinol suggests that certain types of as-yet undefined neoblasts [41] deposited in the canal region might differentiate into diverse cell types during metamorphosis and maturation of the adult worm, during which retinol might mediate essential signaling. This intriguing issue awaits future studies.

Multiple proteins belonging to the iLBP family contain non-classical nuclear localization signal, which is manifested in their folded state, to mediate the nuclear localization of corresponding proteins [37]. The signatures for nuclear localization signal, nuclear export signal and hormone-sensitive lipase binding site together with their regulation sites were also tightly conserved in TsMFABPs (Figure 1A and Figure S1). Interestingly, aa residues comprising the three-dimensional nuclear localization signal are also present in TsMFABPs, which are believed to function in the extranuclear regions as well [37]. These observations suggest that the conformation of nuclear localization signal are distinct along with the respective iLBP:ligand complexes. Subtle shift induced in the tertiary structure of TsMFABP2 by retinol loading might result in the appearance of the recognizable nuclear localization signal and following nuclear translocation to activate genes involved in cellular proliferation and differentiation.

Based on the crystal structures of iLBP:ligand complex, several molecular determinants such as specific aa residues participating in hydrogen-bond interaction(s) with a substrate and in the formation of specific triad structures have been recognized in CRBP and FABP/CRABP family members [42 and references therein]. The size of the binding pocket, which is located inside the β-barrel, is important to determine differential binding activity toward FA ligands [14], [43]. The tertiary structures of TsMFABPs simulated by homology modeling hardly allowed us to recognize significant difference between these proteins, except for two loop-like domains of TsMFABP2 near the bottom of the β-barrel (Figure 1A and Figure S1). The conformation of the extra loops could not be properly simulated by molecular modeling, which made it difficult to predict their actual effect(s) upon binding specificity of TsMFABP2, although one of them contained trp residue, which is known to play an essential role in the ligand binding property by providing a rigid space for the hydrophobic interaction [26]. A recent study with *Caenorhabditis elegans* FA and retinoid binding (FAR) protein exhibited that retinol binding activity is modulated by aa residues lining the ligand binding pocket through casein kinase II phosphorylation [43]. TsMFABP2 harbored three sites for casein kinase II phosphorylation, while TsMFABP1 contained single site. In order to address whether the difference in casein kinase II phosphorylation and presence of two additional loop-like structures are critical for the retinol binding affinity of TsMFABP2, studies employing mutated proteins are currently underway.

Unlike in deuterostomians, divergence of FABP-like proteins into each of the subfamilies seemed not yet occurred in protostomians including parasitic cestodes (Figure 1B and Figure S2). Considering the fact that all the vertebrate iLBP genes conserve their genomic structures composed of four exons and three intervening introns [13], each gene for the subfamily lineages might have duplicated during an early stage of chordate evolution [41]. Meanwhile, invertebrate homologs display exon-intron structures distinguishable along with their donor organisms [13]. The differentially conserved genomic structures are also observed among cestode orthologs [24]. The *S. japonicum* FABP genes appeared to have lost an intron [22]. These collective data suggest strongly that the iLBP family genes have undergone structural remodeling processes such as gain and loss of intron, each of which is rather lineage-specific, in diverse lower animals. Genomic structures of *TsMFABPs* were identical to those of *E. granulosus*, although the lengths of the intron and 3′-untranslated region as well as that of the first exon were significantly enlarged in *TsMFABP2*. Therefore, *TsMFABP2* might have been subject to undergo selection pressure to maximize tolerance against intragenic transposition of nucleotide fragment(s), which can influence the coding profile and/or expression pattern of the related gene.

Genomes of the cestode including TsM might encode multiple iLBPs to maintain metabolic homeostasis and to ensure their long-standing survivals in the unfavorable host environments [10], [11], [23], [24]. *E. granulosus* also expressed at least two distinct FABPs (EgFABP1 and 2; sequence identity with 75%), which showed structural topology and ligand binding activity comparable each other [23], [26]. The reason why these helminths express multiple proteins with similar structural/biochemical properties has not yet been appropriately addressed. Nevertheless, it seems apparent that the paralogous genes have undergone or are undergoing structural diversification processes such as extension of coding region, which eventually lead to functional divergence.

Our results suggest strongly that divergent biochemical properties and physiological roles of the TsM iLBPs might be one of the critical mechanisms compensating for inadequate *de novo* FA synthesis. Further identification of active regulatory elements and related triggering molecules to induce TsMFABP expressions, together with the biological significance of the extra loops observed in TsMFABP2, may elucidate the individual roles of these proteins in the host-parasite relationships, and parasite growth and development. Identification of such a bioactive molecular system inherent to parasitic cellular homeostasis may contribute to further target novel drugs to control and manage NC.

### GenBank Accession numbers

HQ259679 (*TsMFABP1*) and HQ259680 (*TsMFABP2*).

## Supporting Information

Figure S1 **Simulated tertiary structures of TsM FABPs.** The theoretical structures of TsMFABP1 (B) and TsMFABP2 (C) were predicted by homology model using the *E. granulosus* FABP1 as a template (A). Nuclear localization signal found at K18/R9, R30/21 and K31/22 were conserved at the corresponding positions, together with its regulation site at F58/62. Hormone-sensitive lipase binding sites recognized at K18/R9 and nuclear export signal at L60/62, V82/L82 and M92/L92 (each for TsMFABP1 and 2) were also detected. The pink boxes in panel C indicate the amino acid extensions (BC and FG loops) found in the primary structure of TsMFABP2.
(TIF)

Figure S2 **Phylogenetic analysis of TsMFABP proteins.** The evolutionary positions of TsMFABPs were predicted against protostomian and deuterostomian homologs by a phylogenetic analysis (Jones-Taylor-Thornton model of molecular evolution with a neighbor-joining algorithm). The bootstrapping values of branching nodes, which were estimated using 1000 replicates of initial input, were marked in each of the corresponding positions. In order to simplify, the subtree connecting the diverse deuterostomian homologs was compressed and marked as deuterostomian intracellular lipid binding proteins (iLBPs).
(TIF)

Figure S3 **Purification of recombinant and native TsMFABPs.** (A) The recombinant proteins were purified from *E. coli* transformants by Ni-NTA affinity column and monitored by 15% reducing SDS-PAGE. Lanes U, uninduced cells; I, induced cells; P, purified fraction. (B) The TsM extracts were fractionated through the gel filtration and following ion exchange chromatography. The purified proteins were separated by 2-DE (pH 6–10) and visualized by Coomassie Brilliant Blue G-250 staining (upper panels). The protein spots were examined by immunoblotting probed with specific mouse antisera against each of the recombinant proteins (lower panels). pI, isoelectric point; *M*_r_, molecular mass in kDa.
(TIF)
