# Supplementary figures and images for: Structural and Binding Properties of Two Paralogous Fatty Acid Binding Proteins of Taenia solium Metacestode
Source: PLoS Negl Trop Dis. 2012 Oct 25;6(10):e1868. doi: 10.1371/journal.pntd.0001868 (PMC3493614; doi:10.1371/journal.pntd.0001868)

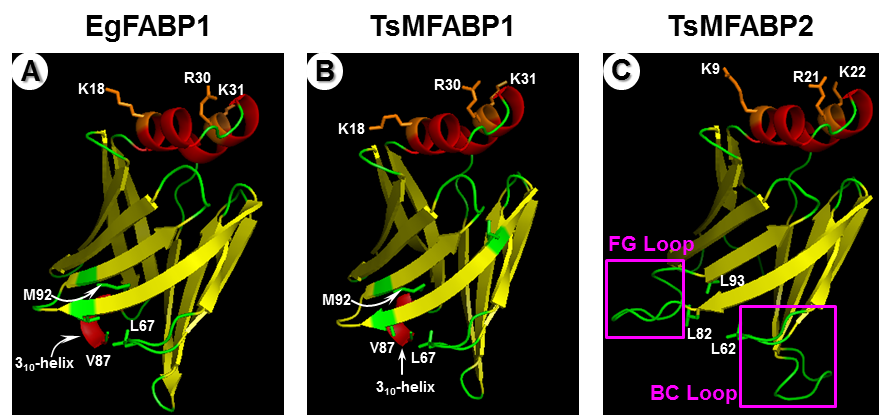

Supplement: Figure S1 — Simulated tertiary structures of TsM FABPs. The theoretical structures of TsMFABP1 (B) and TsMFABP2 (C) were predicted by homology model using the E. granulosus FABP1 as a template (A). Nuclear localization signal found at K18/R9, R30/21 and K31/22 were conserved at the corresponding positions, together with its regulation site at F58/62. Hormone-sensitive lipase binding sites recognized at K18/R9 and nuclear export signal at L60/62, V82/L82 and M92/L92 (each for TsMFABP1 and 2) were also detected. The pink boxes in panel C indicate the amino acid extensions (BC and FG loops) found in the primary structure of TsMFABP2. (TIF) [file pntd.0001868.s001.tif]

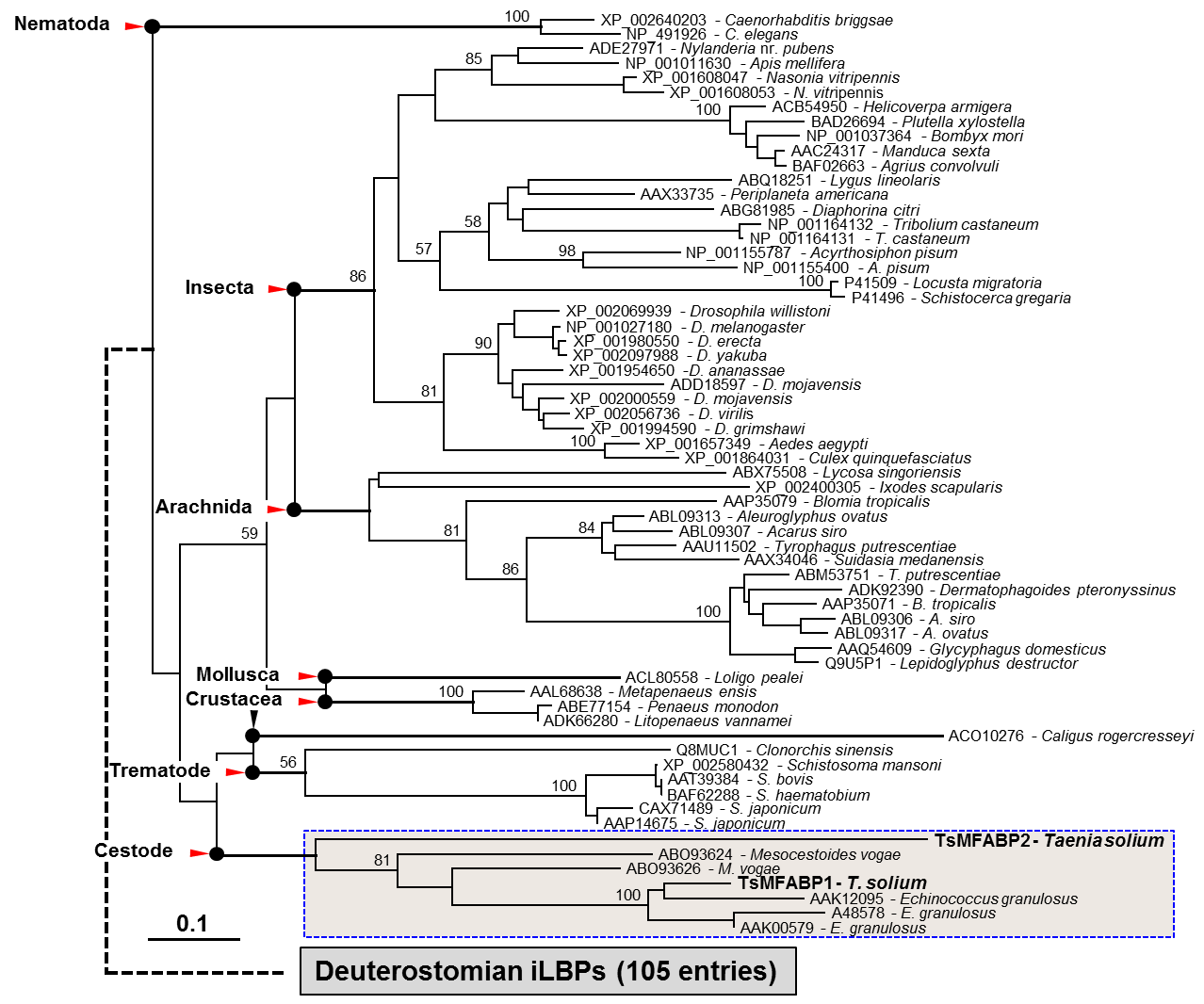

Supplement: Figure S2 — Phylogenetic analysis of TsMFABP proteins. The evolutionary positions of TsMFABPs were predicted against protostomian and deuterostomian homologs by a phylogenetic analysis (Jones-Taylor-Thornton model of molecular evolution with a neighbor-joining algorithm). The bootstrapping values of branching nodes, which were estimated using 1000 replicates of initial input, were marked in each of the corresponding positions. In order to simplify, the subtree connecting the diverse deuterostomian homologs was compressed and marked as deuterostomian intracellular lipid binding proteins (iLBPs). (TIF) [file pntd.0001868.s002.tif]
